# Pebax^®^ 2533/Graphene Oxide Nanocomposite Membranes for Carbon Capture

**DOI:** 10.3390/membranes10080188

**Published:** 2020-08-15

**Authors:** Riccardo Casadei, Marco Giacinti Baschetti, Myung Jin Yoo, Ho Bum Park, Loris Giorgini

**Affiliations:** 1 Department of Civil, Chemical, Environmental and Material Engineering (DICAM), University of Bologna, Via Terracini 28, 40131 Bologna, Italy; riccardo.casadei11@unibo.it; 2 Department of Energy Engineering, Hanyang University, Seoul 133-791, Korea; ymj19@hanyang.ac.kr (M.J.Y.); badtzhb@hanyang.ac.kr (H.B.P.); 3 Department of Industrial Chemistry “Toso Montanari”, University of Bologna, Viale del Risorgimento 4, 40136 Bologna, Italy; loris.giorgini@unibo.it

**Keywords:** Pebax^®^ 2533, graphene oxide, nanocomposite, gas separation membranes, carbon capture

## Abstract

In this work, the behavior of new GO-based mixed matrix membranes was tested in view of their use as CO_2_-selective membrane in post combustion carbon capture applications. In particular, the new materials were obtained by mixing of Pebax^®^ 2533 copolymer with different types of graphene oxide (GO). Pebax^®^ 2533 has indeed lower selectivity, but higher permeability than Pebax^®^ 1657, which is more commonly used for membranes, and it could therefore benefit from the addition of GO, which is endowed with very high selectivity of CO_2_ with respect to nitrogen. The mixed matrix membranes were obtained by adding different amounts of GO, from 0.02 to 1% by weight, to the commercial block copolymers. Porous graphene oxide (PGO) and GO functionalized with polyetheramine (PEAGO) were also considered in composites produced with similar procedure, with a loading of 0.02%wt. The obtained films were then characterized by using SEM, DSC, XPS analysis and permeability experiments. In particular, permeation tests with pure CO_2_ and N_2_ at 35°C and 1 bar of upstream pressure were conducted for the different materials to evaluate their separation performance. It has been discovered that adding these GO-based nanofillers to Pebax^®^ 2533 matrix does not improve the ideal selectivity of the material, but it allows to increase CO_2_ permeability when a low filler content, not higher than 0.02 wt%, is considered. Among the different types of GO, then, porous GO seems the most promising as it shows CO_2_ permeability in the order of 400 barrer (with an increase of about 10% with respect to the unloaded block copolymer), obtained without reducing the CO_2_/N_2_ selectivity of the materials, which remained in the order of 25.

## 1. Introduction

In the last decades, the worldwide rise in energy demand has increased the consumption of fossil fuels, leading to remarkable and regular emissions of combustion products—most of all, carbon dioxide—which, in turn, have led to global warming issues. CO_2_ is indeed the most problematic among the greenhouse gases, not because of its global warming potential [1], but because of the huge amount emitted daily into the environment [2,3].

It is, today, well known that the CO_2_ concentration in the atmosphere has to be reduced to contain the temperature increase below +2 °C compared to the pre-industrial age, as needed to minimize the negative effects of global warming on the planet [4]. To reach this goal, however, the undergoing energy transition towards more green and sustainable sources has to be coupled with more immediate solutions such as the use of the so-called “carbon capture” technologies [5,6] which aim at recovering the emitted CO_2_ and to store it in safe places, thus reducing the CO_2_ emission in the medium–short term.

Nowadays, the most advanced and mature carbon capture technique is solvent absorption [7,8,9], but more environmentally friendly approaches have also been studied, such as mineralization [10], cryogenic separation [11] and, as in this work, membrane separation [12,13,14,15,16].

The main features of gas separation membranes are the permeability of the target compound, in the present case, CO_2_, and its selectivity with respect to other gases present in the stream to be treated. In case of the post combustion approach, where CO_2_ is recovered from flue gas, the main competitor is usually nitrogen.

The permeation through dense membranes is described by the solution-diffusion model, which considers the process as formed by a series of steps involving gas solubilization into the high-pressure side of the membrane, its diffusion across the thickness and, finally, the desorption at the opposite, low-pressure side of the system [17]. Polymer membranes can be very efficient in gas separation, but they present an intrinsic performance limitation as the higher selectivity is usually coupled with lower permeability and vice versa [18].

In order to overcome this tradeoff, several approaches have been considered, such as the use of “mixed matrix membranes” (MMM) which, indeed, have been intensively studied in recent years [16,19,20,21,22,23,24]. In MMMs, different types of organic or inorganic nanofillers are included in the main polymeric matrix in order to provide high selectivity and, possibly also, high permeability.

Among the different polymer considered as starting point, Pebax^®^ materials have been often chosen for their good balance among mechanical resistance, processability, and separation performances [25,26,27,28,29]. Pebax^®^ is the trade name of a series of block copolymers composed by polyamide and polyether blocks, produced with various types of each moiety and in different ratios [30,31,32,33]. They are widely used as a membrane matrix in carbon capture because some of them possess both good CO_2_ permeability and CO_2_/N_2_ selectivity, properties that can be tuned with chemical modification or by the use of fillers, as also reported in the very complete summary from Ho et al. [15].

While the type of Pebax^®^ more commonly used in carbon capture membrane applications is Pebax^®^ 1657, made of nylon-6 and PEO with a respective ratio of 40/60 in mass, other materials of the family also possess interesting performances, such as Pebax^®^ 2533, which was employed in this work as matrix due to the higher CO_2_ permeability showed with respect to Pebax^®^ 1657 (about 130–351 barrer against 45–80 barrer) although with lower CO_2_/N_2_ selectivity (about 25–34 against 43–80) in experiments conducted in mild conditions (pressure upstream of 1–2 bar and temperature from 20 to 35 °C) [27,28,29,34,35,36,37,38,39,40].

Among the many possible fillers usually considered, GO-based nanomaterials have recently attracted much interest and have been widely employed in carbon capture for their capability of enhancing membranes’ gas permselective properties and chemical tunability, with many examples already available in literature [41,42,43,44,45,46,47] and, also, described in reviews such as the one about 0D and 2D filler MMMs by Janakiram et al. [48], or the one specific for graphene and graphene oxide materials from Yoo et al. [49], or even the wider and more general review by Sun et al. [50]. Among the many available studies, one can recall the work from Anastasiou et al. [46], which increased both polysulfone’s CO_2_ permeability (from about 0.9 to 1.8 barrer) and CO_2_/N_2_ selectivity (from about 0.8 to 5) by using a combined system ZIF-8 plus graphene oxide as nanofiller. Similar results improvements were obtained by Dong et al. [44] who managed to increase both CO_2_ permeability (from about 60 to 120 barrer) and CO_2_/N_2_ selectivity (from about 60 to 100) of Pebax^®^ 1657 employing high loadings of porous reduced graphene oxide, or Karunakaran et al., who increased CO_2_/N_2_ selectivity of a PEO–BPT matrix of almost 50%, from 52 to 73, by adding only 0.0625% graphene oxide [51].

As already investigated by Karunakaran et al., GO-loaded membranes tested did not follow the kinetic diameter trend in term of permeation, revealing that the improvement were not due to a physical sieving effect, but to a higher affinity and thus absorption of CO_2_ to GO, as also demonstrated by Shen et al. [52]. That considered, it has to be noted that, generally, the permeabilities of all gases tend to decrease with GO addition, and CO_2_/N_2_ selectivity was incremented by the smaller decrement of CO_2_ itself compared to nitrogen and other gases.

Based on these results and in view of the positive results obtained on Pebax^®^ 1657, the present work aimed at developing a matrix–filler compatibilization procedure by using GO and Pebax^®^ 2533 endowed with higher permeability, but lower selectivity, than Pebax^®^ 1657, with the aim of increasing its selectivity. A recent and accurate economic analysis, provided by Roussanaly et al. [53], about membrane separation in carbon capture showed that a competitive post combustion membrane should to have high permeability coupled with a CO_2_/N_2_ selectivity of at least 100.

Following this idea, in the present work, a set of Pebax^®^ 2533–GO mixed matrix membranes have been prepared and tested to study their potential application for CO_2_ separation in post combustion carbon capture. Different loadings of unmodified GO, used as benchmark filler, were initially considered to optimize the MMM composition. Afterwards, also, porous graphene oxide (PGO), which has similar structure of GO but is less polar and with higher surface/volume ratio [44,54], and graphene oxide functionalized with polyetheramine (PEAGO), which should have a better polymer and CO_2_ compatibility, were used [55].

## 2. Materials and Methods

**Figure 1:**
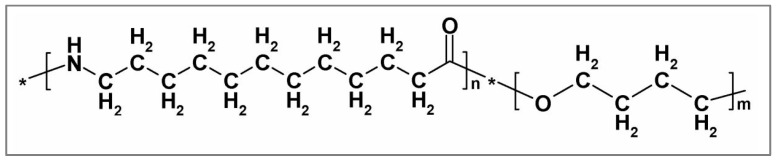
Pebax^®^ 2533 chemical structure.

Pebax^®^ 2533, is a block copolymer of the Pebax^®^ thermoplastic elastomer family produced and provided by Arkema S.r.l. It is made of two blocks: one of polyamide-12, which occupies 20% of the copolymer mass, while the remaining 80 wt% is polytetramethyleneoxide, as also confirmed by many studies found in the literature [56,57,58,59,60]. The structure of Pebax^®^ 2533 is shown in Figure 1.

During the procedure, different solutions and solvents have been used: ethanol, acetone, hydrochloric acid, and sodium hydroxide were provided by Dae-Jung Chemicals, 1-ethyl-3-(3-dimethylaminopropyl)carbodiimide hydrochloride (EDC) and N-hydroxysuccinimide (NHS) by Sigma Aldrich (city, state abbr. if USA or CA, country), and graphite (used to prepare GO and the other GO-based materials) by Bay Carbon Inc. (Bay City, MI, USA).

### 2.1. Nanofiller Preparation

#### 2.1.1. Graphene Oxide

Graphene oxide (GO), synthesized daily in the laboratory of professor Park, was prepared using modified Hummer’s method [61] by stirring 10 g of highly pure graphite powder in 380 mL of H_2_SO_4_ at 5 °C for 10 min, and adding 50 g of KMnO_4_ and stirring for 12 h at 35 °C. Then, the dispersion was highly diluted by adding first 500 mL at 5 °C while stirring for 1 h, then another 2 L of water. The last oxidative step was performed by adding H_2_O_2_ dropwise until the dispersion color changed from dark brown to golden yellow.

Immediately after the synthesis, the so-obtained GO was stirred for 30 min and then filtered, and washed multiple times with 10% HCl and acetone in order to remove any residual impurities [62].

#### 2.1.2. Porous Graphene Oxide

Porous graphene oxide (PGO) was also available in the professor Park’s laboratory, where it is normally synthesized through the following standardized procedure. A 2 mg/mL GO dispersion in water is sonicated for 30 min to ensure high homogeneity. The dispersion was then heated up to 50 °C and NH_4_OH and H_2_O_2_ were added with a volume ratio of GO/NH_4_OH/H_2_O_2_ = 20/1/1. The solution was stirred at constant temperature for 5 h, and then cooled and centrifuged for 1 h at 12,000 rpm to recover the precipitated PGO. This was then redispersed in DI water and dialyzed for 3–4 days to eliminate any residual reagents or impurities. During the dialysis, the pH was continuously monitored, and the process was completed when the solution reached neutrality (pH = 7) [63].

#### 2.1.3. Graphene Oxide Functionalized with Polyetheramine

Graphene oxide functionalized with polyetheramine (PEAGO) was prepared using the method designed by Yoo et al. [55]: 200 mg of 1 mg/mL GO/water dispersion was mixed with 150 mg of Jeffamine M-1000 and stirred for 24 h at room temperature, the pH was then adjusted to 4.5 using 1 M HCl, and 200 mg of EDC and NHS were added to the mixture. The solution was sonicated for 30 min and then stirred for 24 h to complete the modification. Dark brown PEAGO so-obtained was finally washed with HCl and then with acetone in order to remove any impurities.

**Figure 2:**
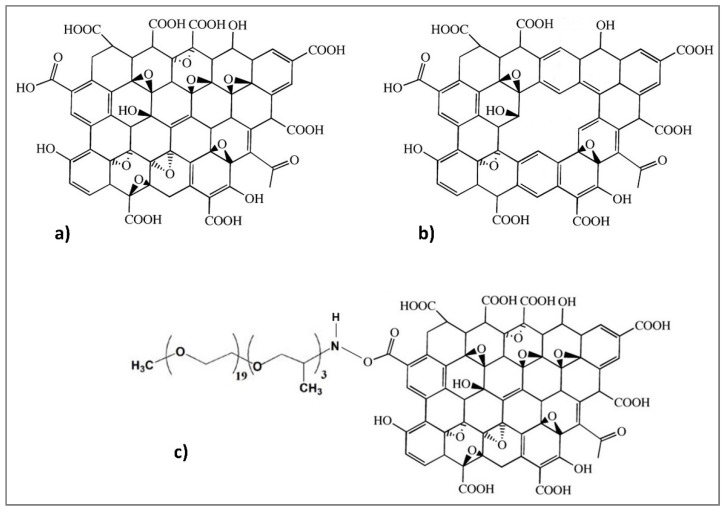
Chemical structures of (**a**) GO [64], (**b**) PGO, and (**c**) PEAGO.

The three GO-based structures are shown in Figure 2:

### 2.2. Membranes Preparation: “Double-Solvent Compatibilization”

Pebax^®^ 2533 and graphene oxide are compatible in different solvents, the former in alcohols (ethanol and, even better, in butanol), the latter just in water. In order to mix them homogeneously and avoid phase segregation, a double-solvent solution has been developed as explained in this section, in order to compatibilize these incompatible materials.

In order to prepare polymeric solutions, Pebax^®^ 2533 pellets have been solubilized in ethanol [26,65] with a concentration of 2 wt% of polymer/solvent by stirring at about 700 rpm upon a 150 °C hot plate for 5 h, and then letting it reach room temperature on 500 rpm stirring.

Water was instead used as dispersive solvent for graphene oxide, which readily precipitated when mixed with ethanol. The water–GO dispersions used in the present work were characterized by a concentration of 0.5 mg/mL and were produced by mixing the GO powder with the desired amount of water and then sonicating the solution for 2 h while maintaining the temperature below 35 °C. The same procedure was used also for the other two types of graphene considered in the present work namely PGO and PEAGO.

**Figure 3:**
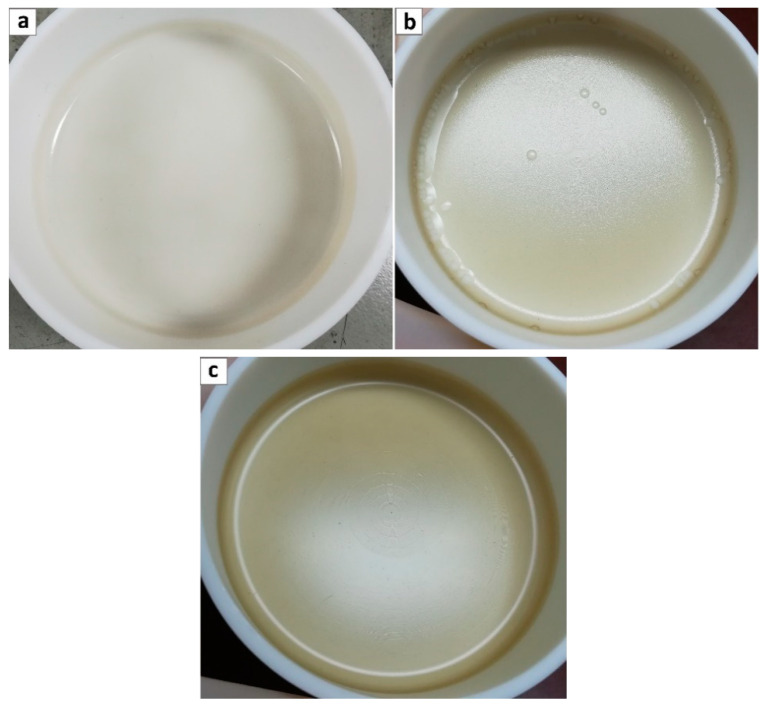
Pebax^®^ 2533 + graphene oxide composite casted membranes; loadings (**a**) 0.1 wt%, (**b**) 0.5 wt%, and (**c**) 1 wt%.

Once the aqueous dispersion of GO was ready, it was added to the Pebax^®^ 2533/ethanol polymeric solution at 35 °C which was then vigorously stirred for 10 min before pouring it in a teflon petri dish. The solution was then left to evaporate at room temperature in order to obtain self-standing membranes. Four different loadings were considered for GO addition, namely 0.02, 0.1, 0.5 and 1 wt%, to study the influence of this parameters on the overall polymer permeability. In all cases, polymer + GO ethanol/H_2_O mixture resulted very homogeneous and allowed obtaining very smooth membranes with no sign of agglomerates even at the highest loading inspected, as shown in Figure 3a–c.

**Figure 4:**
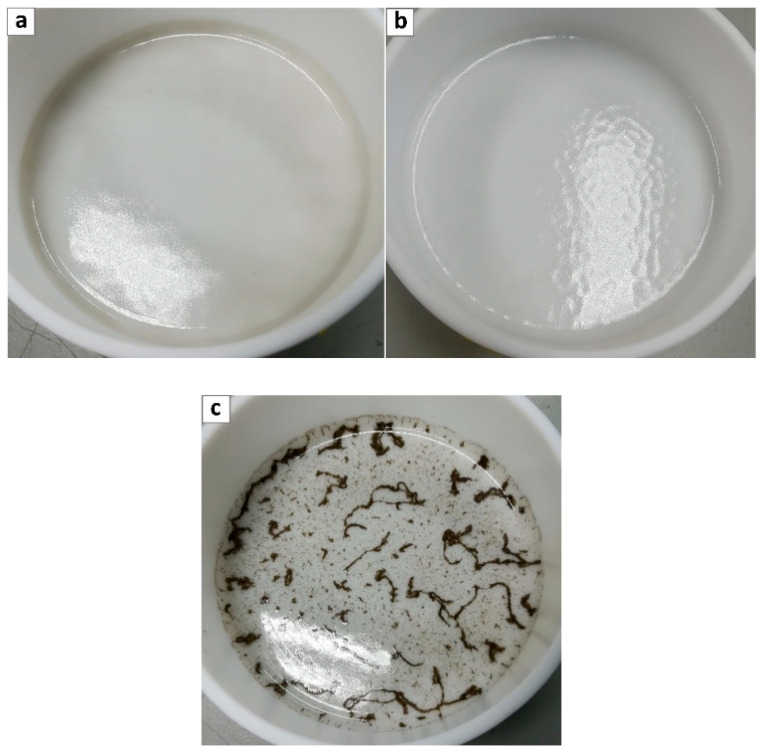
Pebax^®^ 2533 loaded with (**a**) 0.02 wt% PGO, (**b**) 0.02 wt% PEAGO, and (**c**) 0.1 wt% PGO.

Unfortunately for the two other GO types considered, the dispersion in Pebax^®^ 2533 resulted in more difficulties, and for both PGO and PEAGO, only the 0.02 wt% loaded films were obtained with good homogeneity and limited presence of GO aggregates (see Figure 4a,b) while increasing concentration always led to aggregation and heterogenous films as shown in Figure 4c.

The thickness of different tested membranes ranged from 75 to 100 µm, and every resulting membrane was very homogeneous, with a variation in thickness of less than 2% on each sample, as determined from a minimum of 6 measurements on different part of the membranes.

### 2.3. Materials Characterization

#### 2.3.1. XPS

XPS analysis was carried out to confirm that PEA groups were successfully grafted onto GO by monitoring the nitrogen peak of the material. The analysis was conducted using an Omicron ESCALAB (Omicron, Taunusstein, Germany) with a monochromatic Al Kα (1486.8 eV) 300 W X-ray source, and a flood gun to counter charging effects under ultra-high vacuum (UHV ≈ 1 × 10^−9^ torr) conditions.

#### 2.3.2. SEM Analysis

In order to have more precise information about filler dispersion, SEM analysis has been conducted considering both planar and fracture surfaces of membranes of pristine Pebax^®^ 2533 and of all composite samples. 

To obtain information on membranes’ surface, flat pieces of every type of tested material was analyzed, while the fracture surfaces were realized by cutting the membranes in liquid nitrogen with scissors. Indeed, even after immersion, it was impossible to obtain a brittle fracture of the materials by simply bending the samples, which remained rather flexible and tough even when immersed in liquid nitrogen.

SEM images have been carried out using a Phenom ProX (ThermoFisher Scientific, Waltham, MA, USA), with a voltage of 10 kV and a magnification range of 1000–5000×. Before the analysis, all samples have been metal-coated in a Quorum sputter SC7620 using an Au–Pd disc target.

#### 2.3.3. DSC

To better understand how the filler may have affected the matrix structure and if there was preferential distribution within the two copolymer moieties, DSC analysis has been carried out as well. The program used was always made of four cycles, always with a heating rate of 10 °C/min:

(1) Cooling from room temperature to −80 °C
(2) Heating from −80 to 250 °C
(3) Cooling from 250 to −80 °C
(4) Heating from −80 to 250 °C

In this way, with cycles 1 and 2, the thermal history of the material was cancelled, and it was possible to obtain information on the tested material by analyzing cycles 3 and 4.

The apparatus employed was a DSC Q2000 (TA Instruments, New Castle, DE, USA), and the sample size was always between 5 and 25 mg.

#### 2.3.4. Permeation Test

**Figure 5:**
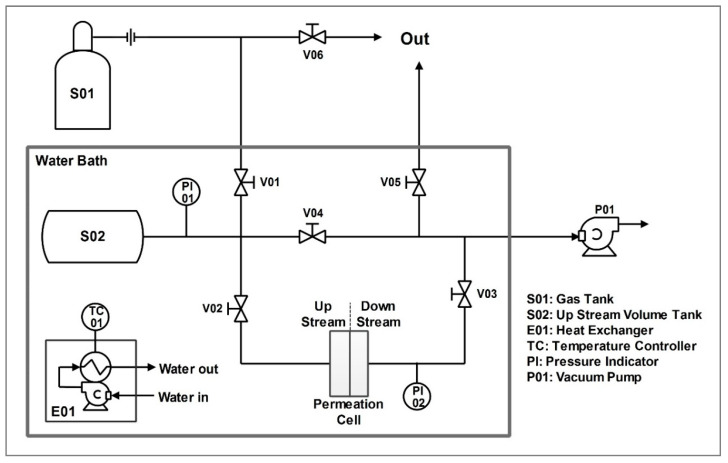
Permeation system employed.

Permeation tests were carried out with the system shown in Figure 5, which has been designed to perform single gas permeability measurement using the so-called barometric technique [24,28]. All experiments have been conducted at 35 °C, with temperature maintained constant by using a heated water bath, which contained almost the entire system. In every experiment, the upstream pressure was maintained at 1 bar while the downstream side was under vacuum conditions.

Technically, the test procedure started by placing the membrane sample into the cell, and then treating it under vacuum until pressure indicators PI01 and PI02 measured a steady value of upstream and downstream pressure. The permeating gas was then charged into the upstream volume (limited between valves V01, V02, and V04, including the tank S02) at the pressure of 1 bar.

Once the upstream pressure was stabilized, the test was started by opening valve V02 while keeping V01, V04, and V03 closed; during the tests, the downstream pressure was continuously monitored and saved to allow then the calculation of permeability (*P*) through Equation (1).

(1) $$P=\frac{V_{d}}{RT}\frac{dP_{d}}{dt}\frac{\delta }{A\Delta p}$$

where pressure Δ*p* indicates the pressure difference between upstream and downstream compartment, *dP_d_/dt* the rate of the downstream pressure increase, *δ* and *A* the membrane thickness and area, respectively, and *V_d_* the downstream volume. All quantities were reported with correct units to obtain the permeability in Barrer (1 Barrer = 10^−10^ (cm^3^_STP_/(cmHg·s·cm)) or 3.35 × 10^−16^ (mol/Pa·s·m)).

All tests were set up to end when an increase of 10 torr in downstream was registered: this value is indeed high enough to allow the measurement of permeability, while not significantly decreasing the pressure driving force between upstream and downstream sections of the apparatus.

Since the system used allowed only one gas to be tested at once, in order to achieve information on membranes’ selectivity, ideal selectivity was calculated through the ratio between the permeability of the two different gases of interest, CO_2_ and N_2_, determined separately as shown in Equation (2):

(2) $$\alpha_{CO_{2},N_{2}}=\frac{P_{CO_{2}}}{P_{N_{2}}}.$$

Overall, the error on the results of gas permeation test, considering thickness variations, volume calibration uncertainty, and other sources of error, was in the order of 2%.

## 3. Results and Discussion

### 3.1. XPS

**Figure 6:**
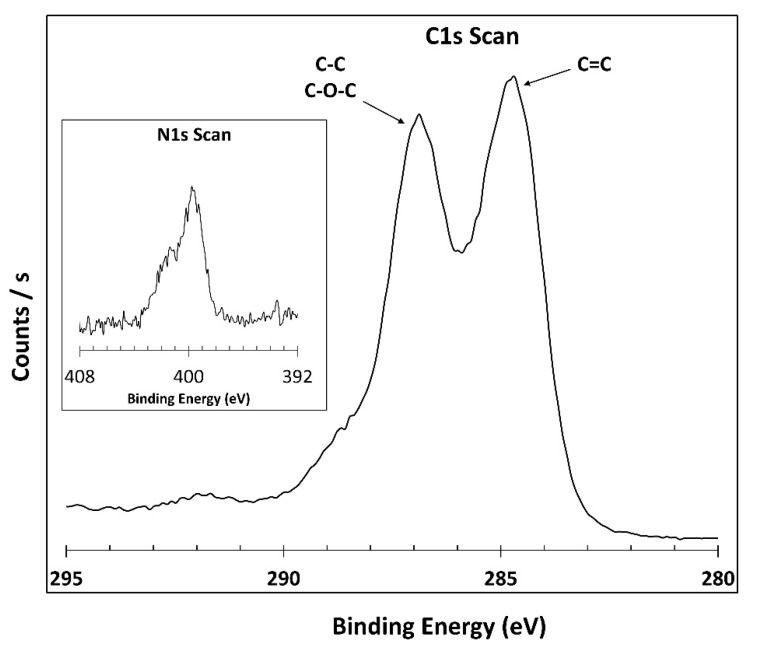
C1s and N1s XPS scan of PEAGO.

To investigate PEAGO reaction and verify the success of grafting, XPS analysis was carried out, focusing on the signal related to nitrogen and carbon and obtaining the results shown in Figure 6.

From the XPS analysis, a small amount of nitrogen 1s was found into the structure, while almost negligible changes were present in the C1s region: PEA already carries a low amount of nitrogen itself, so a high amount of this atom is not expected after the reaction with a nitrogen-less material like GO. XPS confirmed the nitrogen presence and therefore ensured the success of the reaction with a conversion similar to the one obtained from Yoo et al. [55] which, indeed, also presented very similar XPS results.

### 3.2. SEM

Through the SEM images presented below, it has been possible to obtain information on the membrane microstructures, which may be helpful to understand material properties, including the permeation behavior.

**Figure 7:**
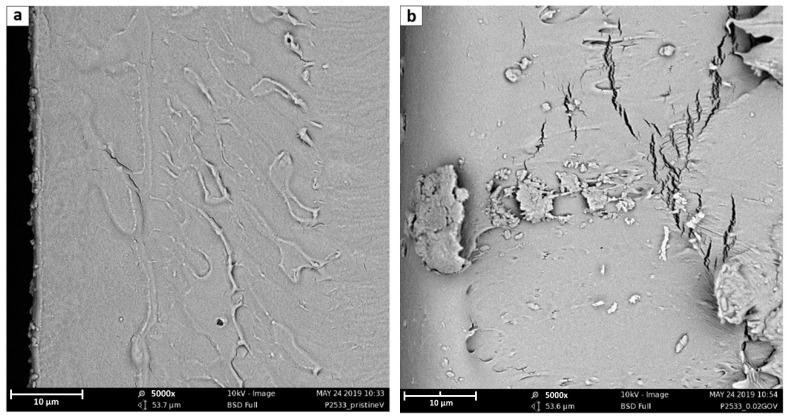

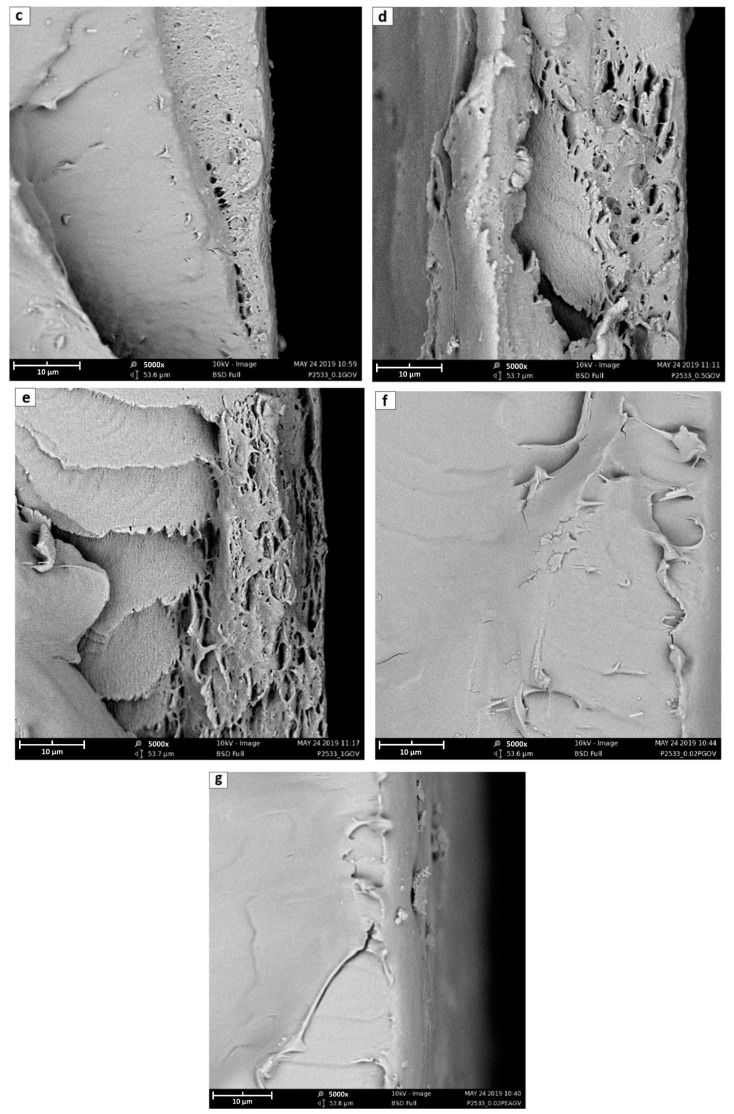
Fracture sections 5000× SEM images of (**a**) neat Pebax^®^ 2533; (**b**) + 0.02 wt% GO; (**c**) + 0.1 wt% GO; (**d**) + 0.5 wt% GO; (**e**) + 1 wt% GO; (**f**) + 0.02 wt% PGO; and (**g**) + 0.02 wt% PEAGO. The scale bar in the figure refers to 10 μm.

Considering the fracture sections, reported in Figure 7, it is possible to notice from that the neat polymer fracture (7a) is the smoothest, while the amount of imperfections increases with the rise of the GO content (7b–e). Indeed, while neat Pebax^®^ shows a very homogeneous structure, the fractured surface of the different mixed matrix membranes always present cracks likely related to nanometric defects, close to the polymer/GO interface, which were amplified during the fracture. Such defects and cracks clearly increase and become more pronounced by increasing the amount of the filler with the 7d and e images, referring respectively to 0.5 and 1% GO loading, that suggest the presence of layered structure on the membrane surface induced by the presence of the filler. These seemed to be characteristic of the lower surface of the membrane suggesting the tendency of the graphene to sediment during the casting procedure; this possibility, however, should be verified further with more specific analyses.

Considering the differences between different types of GO considered, it is worthwhile to note that comparing Figure 7b,f,g, which refer to GO, PGO, and PEAGO with 0.02%wt loading, the latter results have less cracks, suggesting a better interaction between the filler and the polymeric matrix. 

**Figure 8:**
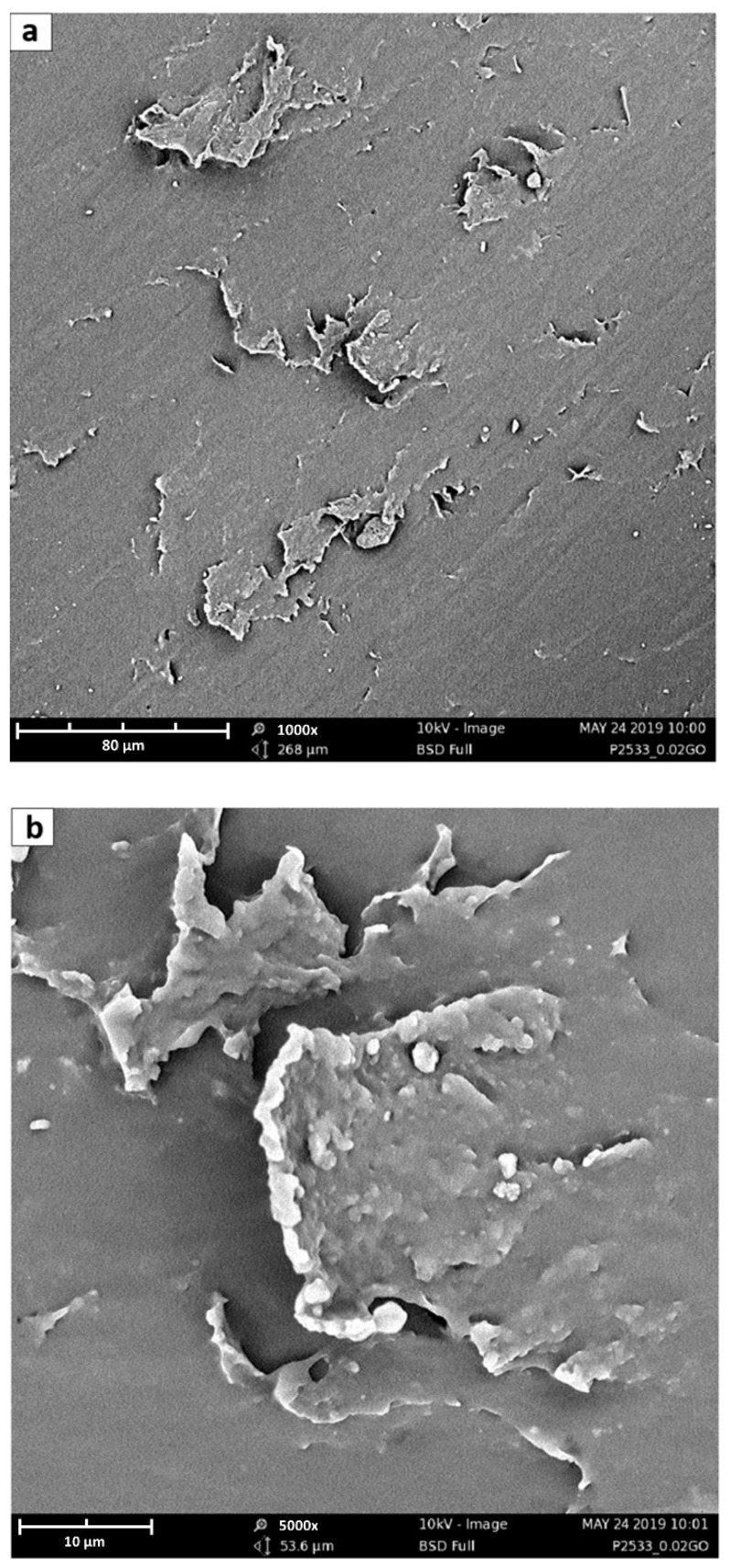
SEM of GO sheets trapped in Pebax^®^ 2533 matrix at (**a**) 1000× and (**b**) 5000×. The scale bars in the two figures (**a**,**b**) refer to 80 and 10 μm respectively.

SEM analysis of the membrane surfaces does not give the same amount of information as the images obtained for the different composites are indeed very similar and are not shown in detail for the sake of brevity. In general, however, they provide indirect information about platelet dimensions; indeed, on membrane surface, it is sometimes possible to see the presence of a high aspect ratio agglomerated GO flakes, partially covered by the polymer. The platelets shown in Figure 8, for example, have lateral dimensions in the order of 10–70 μm, although with the GO synthesis method described in Section 2.1, it is possible to obtain a wide range of GO sizes from hundreds of nanometers to some micrometers, as demonstrated by Cho et al. [66].

### 3.3. DSC

**Figure 9:**
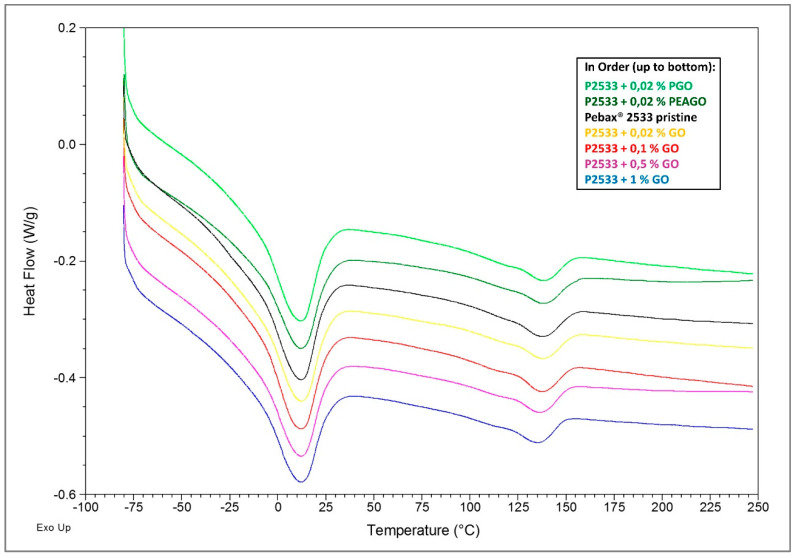
DSC results—4th cycle, heating.

**Figure 10:**
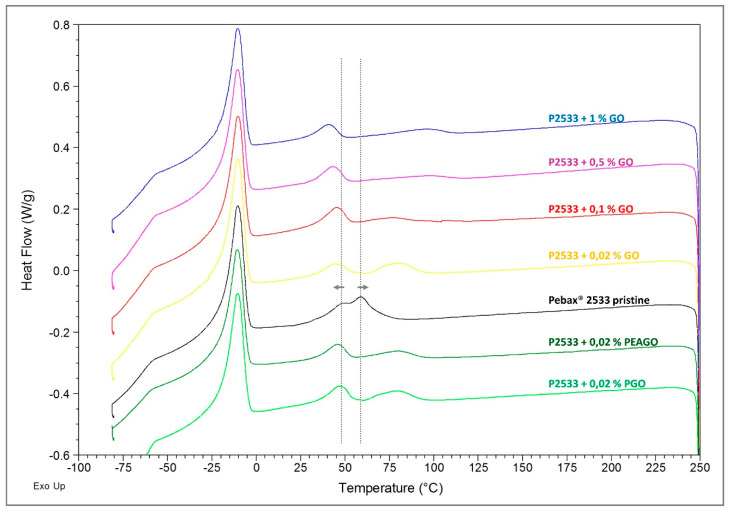
DSC results—3rd cycle, cooling.

DSC analysis was conducted through the four cycles explained in Section 2.3.3, and below are listed the full spectra of the 3rd cycle of heating (Figure 9) and, on the 4th cycle, of cooling (Figure 10), both with shifted lines for the sake of clarity.

In Figure 9, the spectra of the last heating cycle are shown, where the results are quite clear in that all the inspected materials substantially present the same behavior. The two melting peaks (at about 130 °C for the polyamide-12 block and at about 10°C for polytetramethyleneoxide (PTMO) domains) are indeed very similar in all the different spectra, both for position and intensity. It seems, therefore, that the presence of GO nanofillers did not affect the amount or distribution of crystals within the copolymers.

However, looking at Figure 10, referring to the cooling cycle, some differences are clear. Indeed, while the crystallization peak of PTMO (at about −10 °C) remained substantially unaltered in every sample, the crystallization signal of nylon, made up of two partially overlapped peaks at about 50 and 60 °C, was subjected to a slight, but noticeable change. The two peaks indeed shifted respectively toward lower and higher temperatures, thus becoming more and more separated as the filler loading was increased. The shift was then independent of the chemical nature of the materials as in Pebax^®^ 2533 loaded with 0.02% GO, two peaks moved at 45 and 80 °C in almost the same positions observed respectively for the PEAGO and PGO composites with the same filler loading.

Such results suggest that the filler was mainly dispersed into the nylon moiety, affecting its crystallization process. Nylon crystals indeed started to form at lower temperatures with respect to the unloaded polymer (as the GO was acting as nucleating agent) and completed their growth at higher temperature likely due to the increased rigidity of the loaded material, which slowed down the overall process. The platelet, however, did not compromise the completion of the crystallization, nor the structure of the crystals since, as stated above, the melting signals (Figure 9) remained substantially unchanged.

### 3.4. Permeation

**Figure 11:**
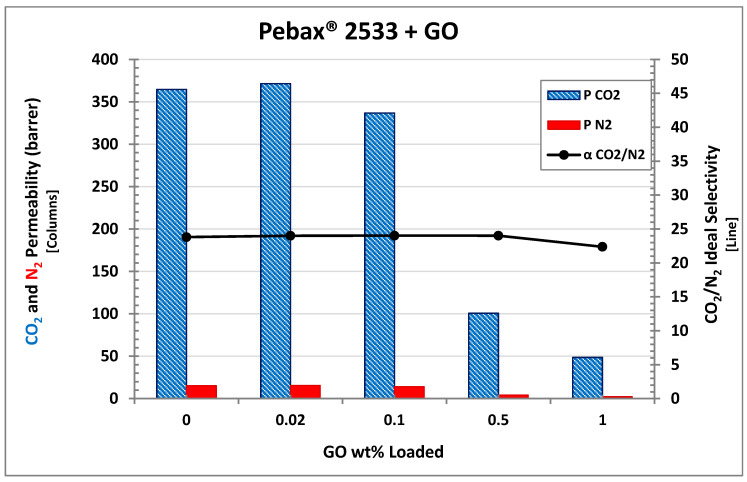
CO_2_/N_2_ permeability and ideal selectivity of Pebax^®^2533 with different GO loadings.

All permeation data measured in the present work are reported in Table 1, which also shows permeability and selectivity variations with respect to pristine Pebax^®^. For a more detailed analysis, Figure 11 shows the results of the permeation tests in the Pebax^®^ 2355 MMMs obtained with different loading of GO. The addition of graphene oxide seems to substantially decrease the permeability for both CO_2_ and N_2_. Indeed from 0.02 to 1 wt% GO, the Pebax^®^-based MMMs show lower and lower permeability with a decrease from 365 to 51 Barrer for CO_2_ and from 15 to 2 Barrer for nitrogen. The only exception in the observed trend is possibly represented by the composite with the lower loading, which indeed showed a very limited increase for CO_2_ permeability (371 Barrer with respect to the 365 Barrer measured for pristine Pebax^®^). Similar behavior was already observed in a previous work made by Lee et al. that were able to improve the permselective properties of polymer matrix Pebax^®^ 1657 loaded with “ZPGO” (that is, ZIF-8 grown on porous graphene oxide), by employing such low filler concentrations [67]. In the present case, however, the difference observed is too limited, within 2%, to conclude that the observed increase is real and not due to an experimental uncertainty, which was also in the order of 2%, considering thickness variation in the membranes and uncertainty in the experimental tests.

**Figure 12:**
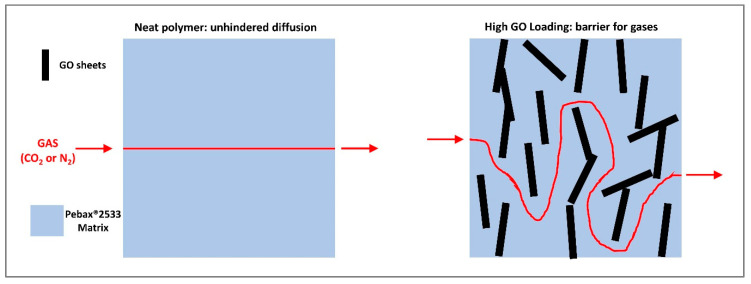
Graphene oxide barrier behavior concept.

The observed decrease in permeability at “high” loading, on the other hand, could be expected as GO platelets can increase the tortuosity of the diffusion path within the membrane, as schematized in Figure 12, due to their high aspect ratio and their low intrinsic permeability, as already reported by other authors [51]. This type of effect seems also confirmed by the SEM images analyzed, where layered structures were clearly visible when the graphene oxide concentration was increased within the matrix.

By comparing the CO_2_ and nitrogen permeability, membrane selectivity can be obtained which is also presented in Figure 11 as a black line. Interestingly, this parameter was substantially unaffected by the addition of the filler, somewhat in contrast with the expected behavior since GO is usually considered to be selective towards CO_2_ and already proved to be able to improve polymer selectivity when used to obtain mixed matrix membranes [41,42,43,44,45,46,47]. 

In the present case, instead, no substantial changes in CO_2_/N_2_ selectivity were observed, as this parameter indeed remained very close to 24 for all the materials tested, so that GO seemed to behave as an impermeable filler which reduced the permeability merely because of tortuosity effects without affecting the permselective behavior of the polymer. 

**Figure 13:**
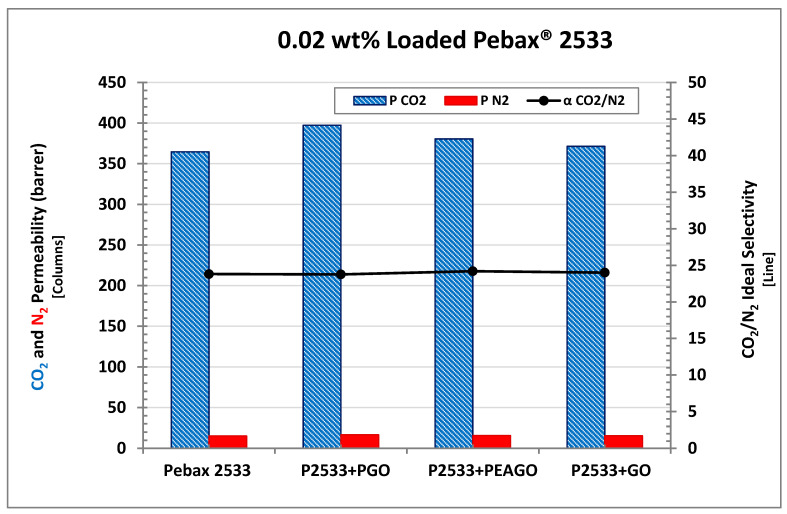
CO_2_ permeability of Pebax^®^ 2533 filled with 0.02 wt% of different GO-based materials.

In order to test different GO-based fillers behavior, Pebax^®^ 2533 membrane mixed with PEAGO and PGO have been also tested, and all of them were compared with 0.02 wt% loading which, based on GO analyses, resulted in being the most promising in terms of permselective properties. The results are shown in Figure 13, where the permeability of CO_2_ and N_2_ in PGO and PEAGO nanofiller are reported together with those already discussed of pristine Pebax^®^ and 0.02 wt% GO membrane that are repeated for the sake of clarity. 

Interestingly, both PEAGO and PGO slightly improved permselective properties with respect to the base polymer with differences between 4% and 8% (that is higher than the recognized experimental error). CO_2_ permeability, in particular, reached the value of 380 Barrer in the PEAGO-loaded sample, and increased even more in PGO-loaded Pebax^®^, where it reached 397 Barrer. CO_2_/N_2_ selectivity, on the other hand, was still unaltered, remaining very close to 24 also for the last two samples inspected.

**Figure 14:**
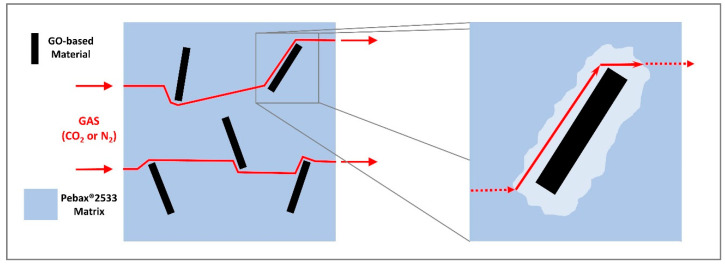
GO material–Pebax^®^ interface voids concept.

Such an increase in permeability without any effect on selectivity cannot be explained by gas platelet interaction which would favor CO_2_ with respect to nitrogen, so that it seems to be related to the presence of a lower density region (possibly related to poor interfacial adhesion or less efficient polymer packing) at the polymer–filler interface where the permeating gases, both CO_2_ and N_2_, can permeate at a higher rate, thus reducing the time to cross the membranes without altering the relative selectivity, as already suggested by various authors [68,69,70,71]. A schematic concept of this hypothesis is displayed in Figure 14.

From this point of view, the performance differences among the three GO materials at the same low concentration (0.02 wt%) are probably related to the dispersion of the different types of GO have in the matrix, which influences both the increase in tortuosity and the properties of the interfacial region. These two processes indeed have opposite effects on permeability, and their balance gives the final materials properties. In the present case, the observed behavior could be explained by considering that, very likely, two modified GOs have lower aspect ratio with respect to the pristine one, due to the additional harsh treatment that the original materials had to undergo to be modified.

Porous graphene oxide is indeed well known to be have reduced area compared the pristine GO and also an higher amount of defects, which makes the nanofiller intrinsically more permeable: Lee et al. [67] demonstrated the formation of pores upon GO, and most likely, such treatment was also able to partially fracture the sheets themselves. On the other hand, fragmentation of GO platelet during PEAGO synthesis can likely be related to the presence of intensive mixing and sonication, which could produce mechanical stresses able to break GO and PEAGO sheets, as demonstrated also by Baig et al. [72].

The difference between PGO and PEAGO could be related to the presence of ether-based polymer chains grafted to the latter, which can lead to a better interaction with the Pebax^®^ matrix, slightly decreasing the interfacial voids compared to PGO and thus causing a smaller effect on permeability.

Finally, the limited changes observed among different materials can be related to the fact that, as suggested by DSC results, the fillers were preferentially dispersed in the nylon moiety, which was less abundant and also less involved in gas permeation compared to PTMO; the features of the latter phase, which is also the main one responsible for the CO_2_/N_2_ selectivity, indeed remained substantially constant in the DSC chart. Thus, by analyzing both DSC and the interface theory previously explained, the obtained permeation results turned out to be consistent.

In general, however, even if slight improvements have actually been obtained, especially with the addition of PGO, the overall permeation properties of the Pebax^®^ 2533-modified GO were not good enough to overcome current state-of-the-art materials, and remained below the 2008 Robeson’s upper bound which, for a CO_2_ permeability of 400 Barrer, requires a selectivity with respect to nitrogen above 50 to be overcome [18]. On the other hand, the results show the possibility to obtain homogeneous dispersion of nanofiller in the polymeric matrix, thus opening the path for further optimization aimed at increasing not only permeability but also CO_2_ selectivity of the obtained MMMs.

## 4. Conclusions

Nanocomposite membranes of Pebax^®^ 2533 have been prepared through its mixing with GO-based materials to investigate the ability of this material to serve as a matrix for MMMs. 

First of all, the behavior of graphene oxide loaded into the polymer at different concentrations was investigated, revealing that 0.02 wt% was the best composition in terms of improvements in properties. Higher loading indeed caused the permeability to drop from 364 barrer of pristine Pebax^®^ 2533 to 50 barrer for the 1 wt% GO loaded sample. SEM analysis revealed indeed that at high concentration, GO platelet tends to aggregate, forming a layered structure with barrier behavior. 

After this initial screening, PEAGO and PGO were also tested at a concentration of 0.02 wt%, showing a further improvement in permselective properties: ideal CO_2_/N_2_ selectivity still remained constant, but CO_2_ permeability increased, even if not dramatically, going from pristine Pebax^®^ (364 Barrer) to the GO (371 Barrer) loaded and then to PEAGO (380 Barrer) and PGO (397 Barrer), which resulted in the most promising nanofiller.

The better performance of PGO may be due to both its sieving behavior, provided by holes on the layer itself, and by its better dispersion caused by the smaller dimensions and surface/volume ratio.

Despite the permeability improvements, the present MMMs still cannot compete with state-of-the-art membrane materials for post combustion carbon capture, and further optimization is needed to also obtain an improvement in the CO_2_/N_2_ selectivity. The current results, in particular, allowed determining the optimal graphene oxide loading, but more attention should be given toward controlling the GO dispersion; a proper control of the distance among platelets, indeed, would hinder the diffusion of nitrogen molecules without substantially affecting the CO_2_ permeability, thus obtaining a molecular sieving structure able to increase the overall membrane selectivity.

## Figures and Tables

**Table 1:** Summary and variation in percentage of permeation results.

Membrane	CO_2_ Permeability (Barrer)	CO_2_ Permeability Variation%	N_2_ Permeability (Barrer)	N_2_ Permeability Variation%	CO_2_/N_2_ Selectivity	CO_2_/N_2_ Variation%
**Pebax 2533**	364.61	\	15.32	\	23.80	\
**+0.02% GO**	371.39	1.86	15.47	1.01	24.00	0.84
**+0.1% GO**	336.80	−7.63	14.02	−8.47	24.02	0.92
**+0.5% GO**	100.61	−72.41	4.19	−72.65	24.01	0.88
**+1% GO**	48.58	−86.68	2.17	−85.83	22.38	−5.97
**+0.02% PGO**	397.35	8.98	16.73	9.19	23.75	−0.19
**+0.02% PEAGO**	380.44	4.34	15.73	2.65	24.19	1.64
